# Phloretin Attenuates Cancer Cachexia-Induced Skeletal Muscle Wasting Associated with the Modulation of STAT3 Signaling

**DOI:** 10.3390/biomedicines14051004

**Published:** 2026-04-28

**Authors:** Kai Lin, Mei-Wei He, Fei Wang, Xin-Yu Hu, Zi-Yue He, Chen-Lu Zhang, Zhi-Qiang Huang, Hong-Wei Wang

**Affiliations:** 1 State Key Laboratory of Analytical Chemistry for Life Science, Medical School, Nanjing University, Nanjing 210093, China; linkai12233@163.com (K.L.); 502023350012@smail.nju.edu.cn (M.-W.H.); fwang@smail.nju.edu.cn (F.W.); 502024350008@smail.nju.edu.cn (X.-Y.H.); 502024350007@smail.nju.edu.cn (Z.-Y.H.); zhang2023chenlu@163.com (C.-L.Z.); 2 Center for Translational Medicine and Jiangsu Key Laboratory of Molecular Medicine, Medical School, Nanjing University, Nanjing 210093, China

**Keywords:** cachexia, phloretin, skeletal muscle, STAT3, muscle atrophy

## Abstract

**Background/Objectives:** Cancer cachexia (CC) is a metabolic syndrome characterized by the progressive loss of skeletal muscle and adipose tissue during tumor progression. Despite its clinical prevalence, effective therapeutic options are currently lacking. Phloretin, a natural flavonoid with potent anti-inflammatory and antioxidant properties, has unclear efficacy against CC. This study investigates the therapeutic potential of phloretin in ameliorating cancer cachexia. **Methods:** Mouse models of CC were established using BALB/c mice implanted with C26 colon carcinoma cells and C57BL/6 mice implanted with Lewis lung carcinoma (LLC) cells. Upon the detection of palpable tumors, phloretin (10 mg/kg) was administered daily via intraperitoneal injection. At the endpoint, hind limb skeletal muscle, inguinal white adipose tissue (iWAT), and hearts were harvested and weighed. Lean body mass was assessed by analyzing the weight of the carcass following the excision of skin, subcutaneous fat, and visceral organs. Gene expression and protein levels in muscle tissues were subsequently quantified. **Results:** Phloretin administration significantly alleviated tumor-induced loss of tumor-free body weight. It effectively preserved skeletal muscle mass in both C26 and LLC cachexia models, while significantly attenuating adipose tissue depletion in the C26 model. In vitro, phloretin treatment mitigated myotube atrophy induced by C26 conditioned medium. Mechanistically, phloretin inhibited STAT3 activation in skeletal muscle. This inhibition suppressed the expression of the E3 ubiquitin ligases MuRF-1 and Atrogin-1. Furthermore, phloretin concurrently modulated the autophagy pathway. **Conclusions:** Phloretin effectively ameliorates cancer cachexia-induced muscle wasting by targeting STAT3-mediated protein degradation and autophagy pathways. These findings suggest that phloretin represents a promising therapeutic agent for the clinical management of cancer-associated cachexia.

## 1. Introduction

Cancer cachexia (CC) is a multifactorial, tumor-associated metabolic syndrome that develops during tumor progression. It is characterized by a sustained loss of skeletal muscle mass, with or without the depletion of adipose tissue [1]. This progressive decline in muscle mass and function presents a significant clinical challenge [2]. While the precise pathological mechanisms of CC remain to be fully elucidated, systemic chronic inflammation has been identified as a primary driver of cachexia-related muscle atrophy [3].This process is largely mediated by pro-inflammatory cytokines, including tumor necrosis factor-alpha (TNF-α), interleukin-6 (IL-6), and interferon-gamma (IFN-γ) [4,5]. The IL-6/Janus kinase (JAK)/signal transducer and activator of transcription 3 (STAT3) pathway is highly upregulated in cachectic patients and drives muscle atrophy through proteolytic pathways [6]. Additionally, the phosphorylation of STAT3 in the skeletal muscle of cachectic patients induces the transcriptional upregulation of Muscle Atrophy F-box (Atrogin-1/MAFbx), Muscle RING-Finger Protein-1 (MuRF-1), and Myostatin [7]. However, there is currently a lack of effective treatment options for cachectic muscle atrophy.

At present, the U.S. Food and Drug Administration (FDA) has not endorsed a universal standard of care for this condition; traditional dietary supplementation and existing pharmacological regimens are largely ineffective at halting or reversing severe tumor-driven weight loss. In routine practice, nutritional support is frequently delivered inconsistently with variable clinical benefit [8]. Glucocorticoids and anti-cytokine therapies may transiently improve appetite and attenuate systemic inflammation, but their substantial adverse-effect profiles strictly limit long-term application [9,10]. In Japan, the ghrelin receptor agonist anamorelin has been approved for treating cancer cachexia. It has been reported to demonstrate clinically meaningful effects, including increases in body weight and improvements in appetite [11]. However, while available data suggest that anamorelin hydrochloride can enhance appetite and increase lean body mass, its long-term effectiveness, impact on functional endpoints, and applicability to different tumor types and patient subgroups require further rigorous validation [12]. Additionally, the complexity of cancer-associated metabolic reprogramming and substantial interindividual heterogeneity further complicate the development of standardized therapeutic paradigms [13,14]. Continued efforts are needed to identify more effective interventions and implement multimodal, integrated management strategies to mitigate this multifaceted metabolic syndrome and improve patients’ quality of life and clinical outcomes.

Phloretin, also known as 2,4,6-Trihydroxy-β-(4-Hydroxyphenyl) Propiophenone, is a lipophilic chalcone mainly found in apple peels [15]. As a dietary flavonoid, phloretin has a variety of biological activities, such as antioxidant activities [16] and anticancer activity [17]. Physicochemically, phloretin is characterized by its low aqueous solubility and high membrane permeability, which facilitates its passive diffusion across lipid bilayers [18]. Despite its efficient cellular entry via passive diffusion and its potent inhibition of specific membrane transporters such as GLUT1 [19], the oral bioavailability of phloretin is significantly limited by extensive first-pass metabolism and rapid glucuronidation in the gastrointestinal tract and liver [20]. In addition to these activities, phloretin has been shown to inhibit the production of inflammatory mediators [21]. Therefore, we hypothesized that phloretin may hold therapeutic potential against cancer cachexia-induced skeletal muscle atrophy.

Through a multidisciplinary strategy integrating in vivo and in vitro approaches, we established two murine cachexia models and C26-conditioned medium (C26-CM)-induced C2C12 myotube atrophy model to investigate the therapeutic efficacy of phloretin and elucidate its underlying mechanisms in the CC-induced muscle wasting. Phloretin administration significantly attenuated CC-induced body weight loss and skeletal muscle atrophy in vivo. Mechanistically, phloretin suppressed STAT3 signaling, thereby inhibiting the activation of both the ubiquitin–proteasome system (UPS) and the autophagy–lysosomal system (ALS) in skeletal muscle. Similarly, phloretin treatment significantly alleviated C26-CM-induced C2C12 myotubes atrophy in vitro. Collectively, these findings suggest that phloretin is a promising therapeutic candidate for CC-associated skeletal muscle wasting.

## 2. Materials and Methods

### 2.1. Reagents and Antibodies

Phloretin (HY-N0142), phosphatase inhibitor cocktails and protease inhibitors were obtained from MedChemExpress (MCE, Monmouth Junction, NJ, USA). The phloretin stock solution was prepared by dissolving the powder in dimethyl sulfoxide (DMSO, Sigma-Aldrich, St. Louis, MO, USA) to a concentration of 100 mM and stored at −80 °C. For in vivo administration, the stock solution was further diluted in a vehicle consisting of 5% DMSO, 40% PEG300, 5% Tween-80, and 50% saline to achieve the desired concentration. Dulbecco’s Modified Eagle Medium (DMEM, 11330500BT) and RPMI 1640 medium (A1451701) were obtained from Gibco (Thermo Fisher Scientific, Waltham, MA, USA). Horse serum (SH30074.03HI) was acquired from HyClone (Logan, UT, USA), and RIPA lysis buffer (P0013B) was from Beyotime Biotechnology (Shanghai, China). For protein analysis and histology, primary antibodies against p-STAT3 (#9145) and STAT3 (#12640) were procured from Cell Signaling Technology (Danvers, MA, USA). Antibodies targeting p-IκBα (AF1870), p-P65 (AB3013), and P65 (AF1234) were obtained from Beyotime Biotechnology. Antibodies against IκBα (10268-1-AP), LC3 (14600-1-AP), P62 (18420-1-AP), and GAPDH (60004-1-Ig) were purchased from Proteintech (Rosemont, IL, USA). Anti-myosin heavy chain (MyHC; MAB4470) antibodies were acquired from R&D Systems (Minneapolis, MN, USA). Primary antibodies against MuRF-1 (sc-32920) and Atrogin-1 (sc-166806) were obtained from Santa Cruz Biotechnology (Dallas, TX, USA). Peroxidase-conjugated secondary antibodies for Western blot were obtained from Fudebio-tech (Hangzhou, China). Fluorescence-conjugated secondary antibodies for immunofluorescence were obtained from Beyotime Biotechnology. The Masson modified IMEB stain kit (K7298) was obtained from IMEB Inc. (San Marcos, CA, USA). Detailed information regarding all primary and secondary antibodies used in this study is summarized in Supplementary Tables S1 and S2.

### 2.2. Animals Studies

All animal procedures adhered to strict institutional ethical guidelines and were adapted from established cachexia protocols [22]. Six-week-old male BALB/c mice and six-week-old male C57BL/6 mice were purchased from GemPharmatech Co., Ltd. (Nanjing, China). The environment was maintained at 21–24 °C under a standard 12 h light/dark cycle, and mice were provided a 7-day acclimation period prior to any interventions. Two different mouse models predisposed to cachexia were used: Colon 26 carcinoma (C26), which induces cachexia in BALB/c mice, and Lewis lung carcinoma (LLC), which induces cachexia in C57BL/6 mice. C26 or LLC cells (5 × 10^5^ cells/mouse) were injected subcutaneously into the mice (*n* = 6 per group). A total of 24 BALB/c and 24 C57BL/6 mice were used in this study. No animals were excluded from the statistical analysis during the experiment. Phloretin (10 mg/kg) was administered via intraperitoneal injection starting on day 9 post-inoculation, a time point when tumors were palpable and cachexia onset typically occurred. This specific dose was selected based on its proven efficacy and safety in previous murine studies [23]. The endpoint for the C26 model was day 15, and for the LLC model was day 18. Upon completion of the experiments, the hind limb muscles, inguinal white adipose tissue (iWAT), and heart were isolated and weighed. The isolated gastrocnemius (GAS) muscles were stripped of surrounding connective tissues, immediately flash-frozen in liquid nitrogen, and stored at −80 °C for subsequent molecular analyses. For subsequent gene and protein quantification, the frozen muscle tissues were mechanically pulverized and homogenized using a tissue homogenizer in the appropriate lysis buffer. Tumor-free body weight was calculated by subtracting the tumor weight from the final body weight.

### 2.3. Cell Culture

The murine colon carcinoma cell line C26 was purchased from CLS Cell Lines Service (Eppelheim, Germany). The Lewis lung carcinoma (LLC) cell line and the C2C12 murine myoblast cell line (CRL-1772) were obtained from the American Type Culture Collection (ATCC, Manassas, VA, USA). All cell lines were authenticated by their respective providers and routinely tested to ensure they were free of mycoplasma contamination prior to use. The mouse C2C12 myoblasts were cultured in DMEM supplemented with 10% fetal bovine serum (FBS, Gibco, Thermo Fisher Scientific, Waltham, MA, USA) and 1% penicillin/streptomycin (P/S, Gibco, Thermo Fisher Scientific, Waltham, MA, USA) as previously described [22]. To induce myotube formation, cells were switched to differentiation medium containing 2% horse serum and 1% P/S for 4–7 days. C26 cells were cultured in RPMI 1640 medium with L-glutamine, containing 10% FBS and 1% P/S. LLC cells were cultured in DMEM with 10% FBS and 1% P/S. All cell lines were incubated at 37 °C in a humidified chamber with 5% CO2. The C26-derived conditioned medium (C26-CM) was collected after 48 h of culture, filtered through a 0.22 μm filter, and stored at 4 °C. The C26-CM was used to induce atrophy in C2C12 myotubes for subsequent experiments, a well-established in vitro model for cancer cachexia [24]. The phloretin powder was first dissolved in dimethyl sulfoxide (DMSO) to prepare a 100 mM stock solution. To ensure that the final DMSO concentration remained below 0.1% (*v*/*v*) and avoid cytotoxicity, the stock solution was further diluted in culture medium to a working concentration of 100 μM for in vitro treatment. After 4–7 days of differentiation, the myotubes were co-incubated with C26-CM and phloretin for 48 h. Control cells were treated with an equal volume of DMSO as a vehicle control. For the quantification of myotube atrophy, the diameters of C2C12 myotubes were measured using ImageJ software(v1.53, The National Institutes of Health, Bethesda, MD, USA). At least 50 myotubes from 10 to 15 random fields per group were analyzed by calculating the average of three measurements along the short axis of each myotube.

### 2.4. Western Blot Analysis

Immunoblotting procedures were adapted from our earlier protocols [14]. Briefly, total protein extracts were prepared from either skeletal muscle or C2C12 myotube samples using RIPA lysis buffer supplemented with protease inhibitor (Beyotime Biotechnology, Shanghai, China). The protein concentration was determined using the BCA Protein Assay Kit (Beyotime Biotechnology, Shanghai, China)**.** The protein extracts were mixed with 5× loading buffer and denatured at 97 °C for 8 min. Equivalent quantities of protein samples were resolved utilizing 12% SDS-PAGE and subsequently electro-transferred onto polyvinylidene fluoride (PVDF) membranes (IPVH00010, Merck Millipore, Tullagreen, Ireland). For the specific detection of low molecular weight proteins (<20 kDa, such as LC3), proteins were transferred to 0.22 μm PVDF membranes (FFP70, Beyotime Biotechnology, Shanghai, China) to prevent protein breakthrough. Blocking was performed by incubating the membranes with 5% bovine serum albumin (BSA, Sigma-Aldrich, St. Louis, MO, USA) for 2 h at room temperature. Subsequently, the membranes were incubated with specific primary antibodies (diluted 1:500–1:2000; detailed antibody information is provided in Supplementary Table S1) overnight at 4 °C. After being washed with TBST three times, the membranes were incubated with peroxidase-conjugated secondary antibodies (FDM007 and FDR007; Fudebio-tech, Hangzhou, China) for 2 h at room temperature. Immunostained bands on the membranes were visualized using the ECL system (Tanon 4600) according to the manufacturer’s instructions. The optical density of the protein bands was quantified using ImageJ software (v1.53, The National Institutes of Health, Bethesda, MD, USA). Target protein expressions were normalized against GAPDH, while phosphorylated proteins were strictly standardized relative to their respective total protein levels.

### 2.5. Grip Strength Measurement

This protocol was adapted from our previous study [22]. A grip strength meter (SA415, Sansbio, Jiangsu, China) was employed to evaluate both forelimb and four-limb grip strength in mice. During the test, each mouse was lifted by the tail and encouraged to grasp a metal grid connected to a digital force transducer. The tail was then steadily pulled backward, and the peak force displayed on the gauge immediately before the mouse released its grip was recorded as the grip strength value. This assessment was repeated five consecutive times for each subject, and the average of the maximum values was calculated to represent the absolute grip strength.

### 2.6. Immunofluorescence

Cells were plated onto sterile glass coverslips placed within 12-well plates coated with poly-D-lysine (0.1 mg/mL, Sigma-Aldrich, St. Louis, MO, USA) and induced to differentiate. For immunostaining, cells were fixed in 4% paraformaldehyde (30 min) and permeabilized with 0.1% Triton X-100 in PBS. Subsequently, they were incubated overnight at 4 °C with anti-MyHC antibody (diluted 1:100 in 1% BSA/PBST). Subsequently, the cells were incubated at room temperature for 1 h with both a fluorescence-conjugated anti-mouse secondary antibody (1:1000) and DAPI (1:1000). Finally, the coverslips were removed from the wells and mounted onto glass slides. Samples were examined and images were captured under an FV10i laser scanning confocal microscope (Olympus, Center Valley, PA, USA). The mean gray value was quantified using ImageJ software by calculating the ratio of integrated density (IntDen) to the area of interest.

### 2.7. Histological and Immunostaining Analyses

Mice were sacrificed, and skeletal muscle tissues were excised and fixed in 4% paraformaldehyde overnight. The tissues were subsequently dehydrated and embedded in paraffin. For histopathological evaluation, 4 μm thick sections were sliced and stained with hematoxylin and eosin (H&E). Masson’s trichrome staining was performed using a Masson Modified IMEB Stain Kit (K7298, IMEB Inc., San Marcos, CA, USA) following the manufacturer’s protocol. For immunofluorescence analysis, paraffin sections were deparaffinized, antigen-retrieved, and incubated with antibodies following previously reported [25]. H&E and Masson’s trichrome-stained sections were examined using a standard light microscope. Immunofluorescence images were captured using an FV10i laser scanning confocal microscope (Olympus, Center Valley, PA, USA). For the quantitative analysis of stained tissue sections, at least 5 randomly selected fields of view per sample were captured and analyzed to ensure representativeness. Quantitative histological analysis was performed using ImageJ software (NIH, Bethesda, MD, USA). The muscle fiber cross-sectional area (CSA) was determined by manually tracing the boundaries of myofibers from H&E-stained sections. The collagen volume fraction (CVF) was calculated from Masson’s trichrome-stained sections by determining the ratio of the blue-stained fibrotic area to the total tissue area. The percentage of centronucleated fibers was calculated by dividing the number of muscle fibers containing centrally located nuclei by the total number of fibers in the respective field of view.

### 2.8. Statistical Analysis

For in vivo studies, n represents the number of biological replicates. For in vitro studies, results are representative of at least three independent biological experiments performed in triplicate. The data were analyzed by SPSS 18.0 statistical software. The measured data are expressed as the mean ± standard error of the mean (SEM). Data normality was assessed using the Shapiro–Wilk test. The measurement data were tested using Student’s *t* test or one-way analysis of variance (ANOVA) for normally distributed data. For multiple comparisons involving more than two groups, ANOVA was followed by Tukey’s post hoc test to determine statistically significant differences between specific groups. We set the significance level at α = 0.05 and *p* < 0.05 was considered statistically significant. Graphical representations were created using GraphPad Prism 5.0.

## 3. Results

### 3.1. Phloretin-Protected C26-Induced Cancer Cachexia Model

**Figure 1:**
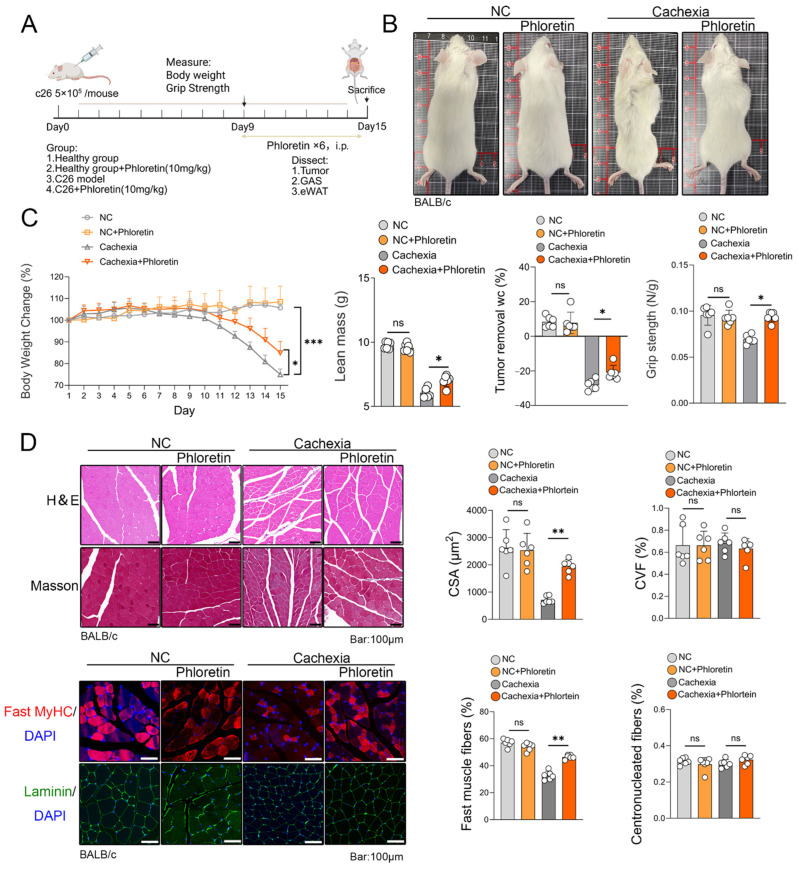
Phloretin protects C26-induced cachexia model. (**A**) On day 9, phloretin was injected into C26 cancer cachexia model mice, and the mice were euthanized on day 15 for tissue collection. (**B**) Phenotypic images of mice. (**C**) Body weight changes, lean mass, grip strength and percentage change in body weight after tumor removal in each group of mice (*n* = 6 in each group). (**D**) H&E staining and Masson staining of the quadriceps femoris muscle reveal the effects of phloretin supplementation on muscle tissue. Immunofluorescence staining of MyHC and Laminin is shown in the left panel, and quantitative analysis of myotube diameter is shown on the right panel. Scale bar represents 100 μm. The data are presented as the mean ± SEM. One-way ANOVA followed by Tukey’s post hoc test was used. Statistical significance: ns means not significant; * *p* < 0.05; ** *p* < 0.01; *** *p* < 0.001.

First, we established a C26 tumor-induced CC model in mice. The animals were treated with phloretin (10 mg/kg, administered daily via intraperitoneal injection) on day 9 and euthanized on day 15 (Figure 1A). As illustrated in Figure 1B, phloretin treatment significantly alleviated cachexia-related physical deterioration. Compared to vehicle-treated mice, cachectic mice exhibited marked emaciation and poor coat condition, both of which were substantially improved by phloretin administration. Furthermore, phloretin treatment markedly reduced body weight loss and increased lean mass in C26 tumor-bearing mice after removing the tumor mass. Grip strength testing showed that phloretin treatment attenuated the reduction in forelimb strength (Figure 1C). Additionally, the mass of iWAT was significantly improved (Supplementary Figure S1A). Collectively, these results demonstrate that phloretin provides systemic protection against CC-induced body weight loss and functional decline.

To investigate whether the observed benefits were linked to reduced skeletal muscle atrophy, we conducted H&E staining, Masson’s trichrome staining, laminin staining and fast MyHC immunostaining. H&E staining and laminin staining of skeletal muscle revealed a significant reduction in muscle fiber cross-sectional area (CSA) in the cachectic skeletal muscle of C26 tumor-bearing mice, an effect that was reversed following phloretin treatment. Masson’s trichrome staining showed no significant improvement in the cross-sectional area fraction (CVF) of muscle fibers following phloretin administration. Additionally, the proportion of fibers with central nuclei did not increase, suggesting that phloretin did not induce substantial muscle regeneration. However, immunostaining for fast MyHC revealed that phloretin treatment increased the abundance of fast-twitch muscle fibers in the cachectic muscle (Figure 1D).

### 3.2. Phloretin Prevents C26-Induced Skeletal Muscle Atrophy by Inhibiting UPS and ALS Activation via the STAT3 Pathway

**Figure 2:**
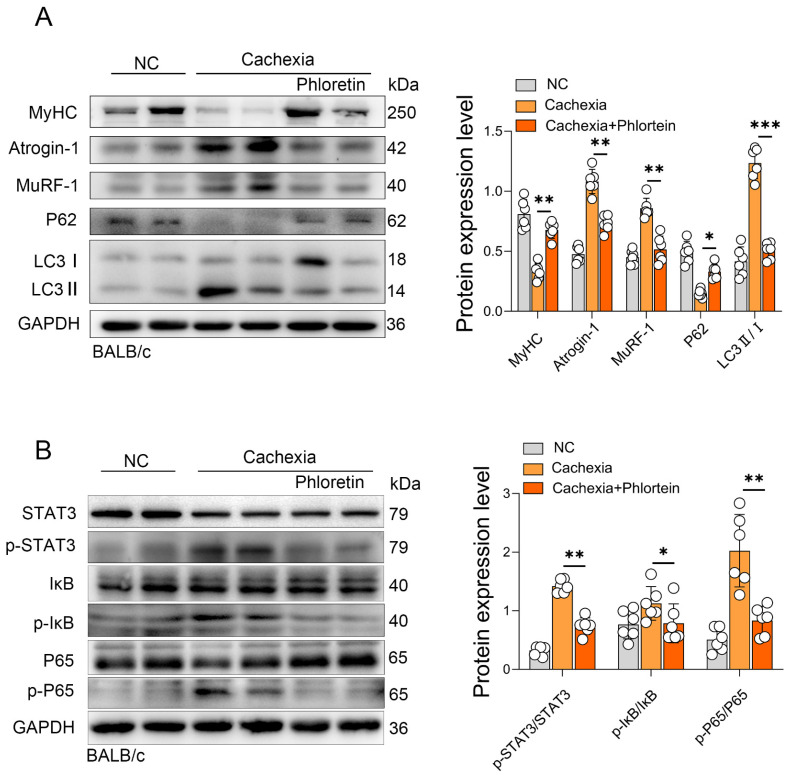
Phloretin prevents C26-induced skeletal muscle atrophy in cancer cachexia by inhibiting the activation of the UPS and ALS through suppression of STAT3 pathway. (**A**) Protein expression of MyHC, Atrogin1, MuRF-1, P62, and LC3 in C26 model mice muscles with phloretin, and quantitative analysis of protein gray scale value on the right panel. (**B**) Protein expression of STAT3, p-STAT3, IκB, p-IκB, P65 and p-P65 in C26 model mice muscles with phloretin, and quantitative analysis of protein gray scale value on the right panel. The data are presented as the mean ± SEM. One-way ANOVA followed by Tukey’s post hoc test was used. Statistical significance: * *p* < 0.05; ** *p* < 0.01; *** *p* < 0.001.

To further elucidate the molecular mechanism underlying phloretin’s protective effect on skeletal muscle atrophy, total protein was isolated from the gastrocnemius (GAS) muscles harvested at the experimental endpoint (day 15). We assessed the activation of the UPS and ALS, the two primary degradation pathways involved in CC-induced skeletal muscle atrophy. Western blot analysis revealed that phloretin treatment effectively prevented the degradation of MyHC. Concurrently, it significantly downregulated the expression of the muscle-specific E3 ubiquitin ligases, MuRF-1 and Atrogin-1 (Figure 2A). Next, we examined the upstream signaling pathways. Our findings revealed that the phosphorylation of STAT3 (p-STAT3) was significantly enhanced in the C26 model, indicating the activation of this pathway under cachectic conditions. However, phloretin treatment significantly reduced p-STAT3 levels. Furthermore, we assessed the NF-κB pathway. We observed enhanced phosphorylation of IκB (p-IκB) and P65 (p-P65) in cachectic muscles, which was significantly attenuated by phloretin treatment (Figure 2B). The results showed that phloretin treatment significantly inhibited the activation of both the STAT3 and NF-κB pathways in C26 tumor-bearing cachectic mice. Additionally, the expression levels of total STAT3, IκB, and P65 normalized to GAPDH were quantified, as shown in Supplementary Figure S2.

### 3.3. Phloretin-Protected LLC-Induced Cancer Cachexia Model

**Figure 3:**
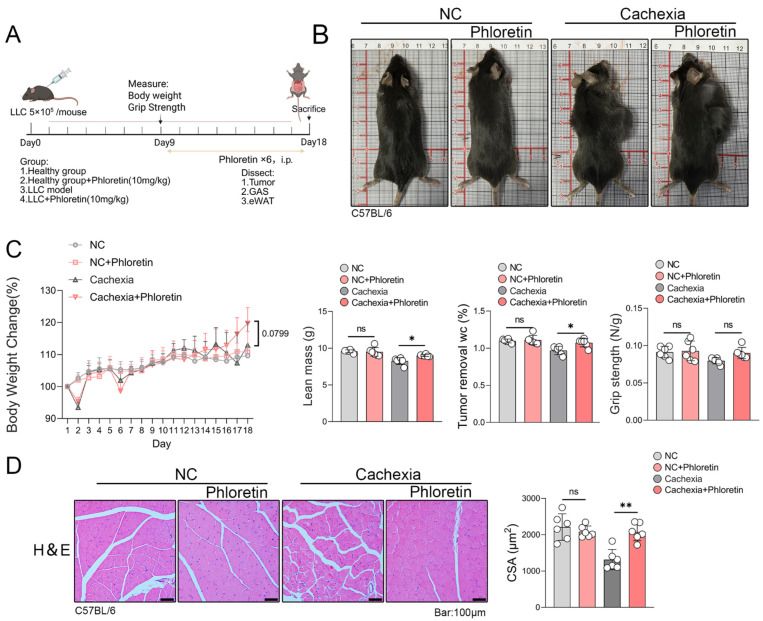
Phloretin protects LLC-induced CC model. (**A**) On day 9, phloretin was injected into LLC cancer cachexia model mice, and the mice were euthanized on day 18 for tissue collection. (**B**) Phenotypic images of mice. (**C**) Body weight changes, lean mass, grip strength and percentage change in body weight after tumor removal in each group of mice (*n* = 6 in each group). (**D**) H&E staining of the quadriceps femoris muscle reveals the effects of phloretin supplementation in the muscle tissue of the LLC model. Quantitative analysis of myotube diameter is shown on the right panel, The scale bar represents 100 μm. The data are presented as the mean ± SEM. One-way ANOVA followed by Tukey’s post hoc test was used. Statistical significance: ns means not significant; * *p* < 0.05; ** *p* < 0.01.

To determine whether phloretin exerts similar effects across different mouse models, we established a model of LLC tumor-induced cachexia. Mice were treated with phloretin injections on day 9 and sacrificed on day 18 (Figure 3A). As shown in Figure 3B, phloretin administration conferred a protective effect against cachexia in the LLC tumor-bearing model. The physical characteristics of cachectic mice, including emaciation, were improved following phloretin treatment, which enhanced their overall body condition. However, due to the slower progression of cachexia in the LLC tumor-bearing model and the large tumor size, the change in body weight was not statistically significant. Phloretin treatment did not lead to a statistically significant improvement in grip strength in the LLC model (Figure 3C), which may be due to the slower progression of cachexia in this model. Finally, to examine the effects of phloretin on skeletal muscle atrophy, we performed H&E staining. The results demonstrated that phloretin significantly alleviated muscle fiber degeneration and increased the CSA of muscle fibers in LLC-bearing cachectic mice (Figure 3D).

### 3.4. Phloretin Prevents LLC-Induced Skeletal Muscle Atrophy in Cancer Cachexia by Inhibiting the Activation of UPS and ALS by Inhibiting STAT3 Pathway Activation

**Figure 4:**
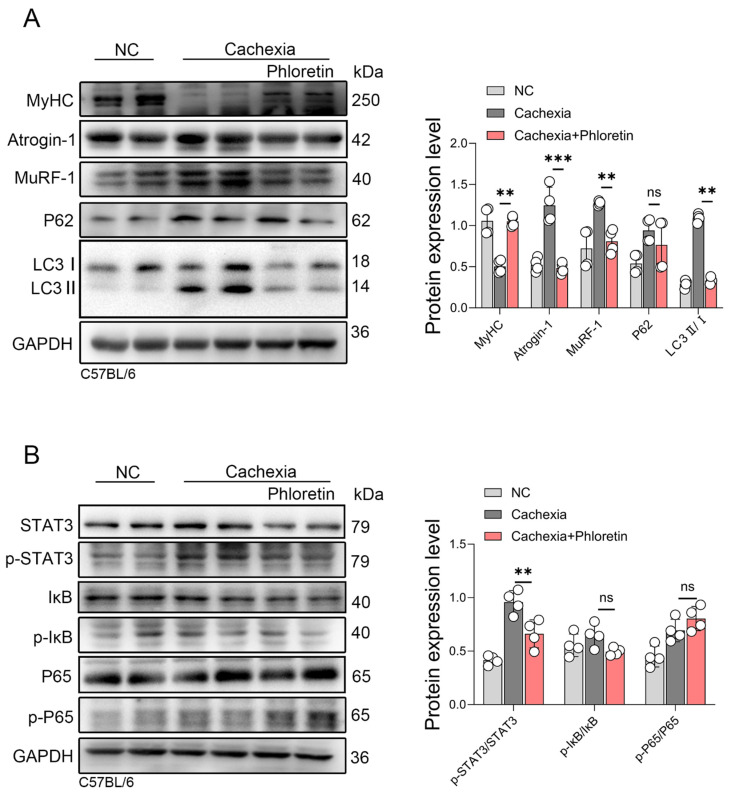
Phloretin prevents LLC-induced skeletal muscle atrophy in CC by inhibiting UPS and ALS activation via suppression STAT3 pathway. (**A**) Protein expression of MyHC, Atrogin-1, MuRF-1, P62, and LC3 in LLC model mice muscles with phloretin, and quantitative analysis of protein gray scale value on the right panel. (**B**) Protein expression of STAT3, p-STAT3, IκB, p-IκB, P65, and p-P65 in LLC model mice muscles with phloretin treatment. Quantitative analysis of protein gray scale value is shown on the right panel. The data are presented as the mean ± SEM. One-way ANOVA followed by Tukey’s post hoc test was used. Statistical significance: ns means not significant; ** *p* < 0.01; *** *p* < 0.001.

To determine whether the protective mechanisms of phloretin are consistent across different tumor models, we analyzed muscle tissues from LLC tumor-bearing mice. The LLC animals received the same treatment regimen as the C26 group, consisting of a daily intraperitoneal injection of phloretin (10 mg/kg) from day 9 to day 18. First, we assessed the UPS. Phloretin treatment prevented the MyHC downregulation. Concurrently, it significantly inhibited the expression of the E3 ubiquitin ligases MuRF-1 and Atrogin-1 (Figure 4A). Next, we examined the ALS. The LC3 II/I ratio was significantly reduced in the treatment group, indicating attenuated autophagy. However, unlike the C26 model, P62 levels did not change significantly (Figure 4A). Finally, we evaluated the inflammatory signaling pathways. Phloretin significantly suppressed the phosphorylation of STAT3 (p-STAT3). Regarding the NF-κB pathway, however, phloretin treatment did not significantly alter the phosphorylation levels of IκB (p-IκB) and P65 (p-P65) (Figure 4B). The corresponding expression changes in total STAT3, IκB, and P65 proteins relative to GAPDH are provided in Supplementary Figure S2. These results suggest that phloretin alleviates skeletal muscle atrophy in the LLC model primarily by inhibiting the UPS and STAT3 pathways.

### 3.5. Phloretin Alleviates the C26-CM Induced C2C12 Myotubes Atrophy In Vitro

**Figure 5:**
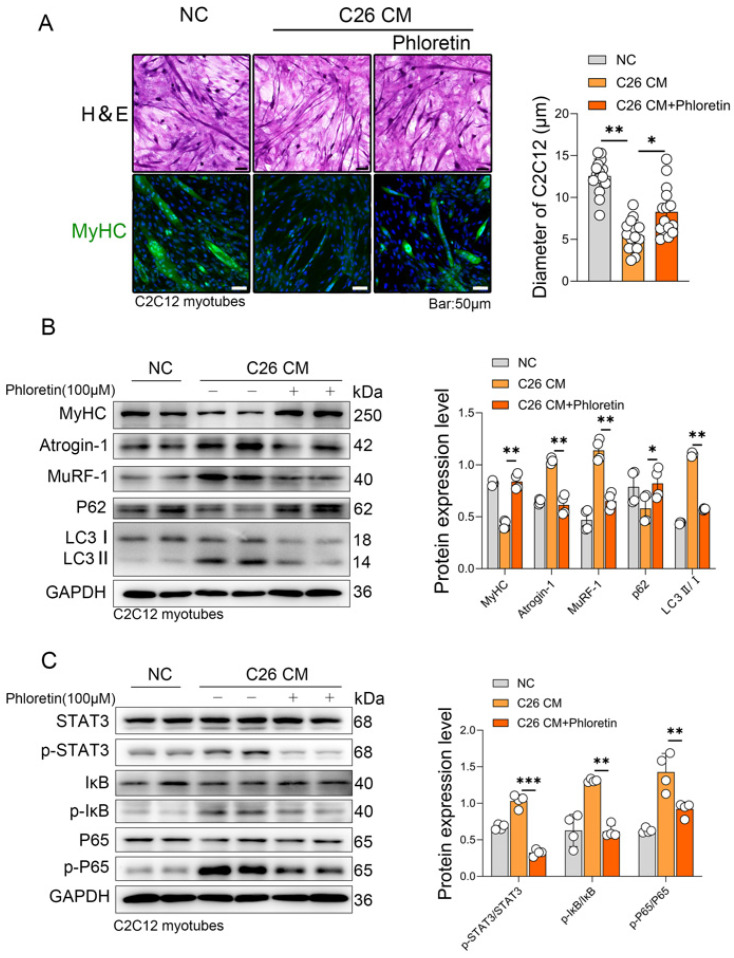
Phloretin alleviated the C26-CM-induced C2C12 myotubes atrophy in vitro. (**A**) H&E staining and immunofluorescence staining of MyHC in C26 cachexia cell model with Phloretin treatment are shown. Quantitative analysis of myotube diameter is shown on the right panel. The scale bar represents 50 μm. (**B**) Protein expression of MyHC, Atrogin1, MuRF-1, P62, and LC3 in C26 cachexia cell model with phloretin, and quantitative analysis of protein gray scale value on the right panel. (**C**) Protein expression of p-STAT3, p-IκB, and p-P65 in C26 cachexia cell model with phloretin, and quantitative analysis of protein gray scale value on the right panel. The data are presented as the mean ± SEM. One-way ANOVA followed by Tukey’s post hoc test was used. Statistical significance: * *p* < 0.05; ** *p* < 0.01; *** *p* < 0.001.

To investigate the role of phloretin in vitro, we used the culture supernatant from the mouse colorectal cancer cell line C26 to induce cachexia in C2C12 myotubes, which served as an in vitro model of cancer cachexia (CM group). Phloretin was dissolved in DMSO to create a 100 mM stock solution, which was then diluted in the culture medium to a final working concentration of 100 μM. The C2C12 myotubes displayed atrophy, characterized by a reduction in diameter and a decrease in MyHC expression. Notably, the atrophy induced by the C26 CM was reversed upon the co-incubation with phloretin (100 μM) for 48 h and MyHC expression was restored as evidenced by immunofluorescence staining (Figure 5A). Western blot analysis revealed that co-culturing C2C12 cells with C26 supernatant led to a decrease in MyHC expression, while the E3 ubiquitin ligases MuRF-1 and Atrogin-1 were significantly upregulated. Additionally, P62 expression decreased, accompanied by an increase in the LC3 II/I ratio (Figure 5B), reflecting an altered autophagic state induced by the C26 supernatant. Mechanistically, both the UPS and ALS pathways were hyperactivated in C2C12 cells upon co-culture with C26 supernatant, and the hyperactivation of these protein degradation pathways was attenuated by phloretin treatment (Figure 5B). Furthermore, we examined the inflammatory signaling pathways. The ratios of phosphorylated to total proteins (p-STAT3/STAT3, p-IκB/IκB, and p-P65/P65) were remarkably elevated in the C26 CM group. Importantly, phloretin treatment significantly inhibited the activation of both the STAT3 and NF-κB pathways by reducing the phosphorylation levels of these proteins relative to their total expression. (Figure 5C). Furthermore, the quantitative analysis of total STAT3, IκB, and P65 relative to GAPDH is presented in Supplementary Figure S2.

## 4. Discussion

CC is a major cause of mortality among cancer patients, accounting for approximately 40% of cancer-related deaths [26]. It is a multifactorial syndrome that is resistant to nutritional interventions [27] and is characterized by severe depletion of skeletal muscle and adipose tissue, features commonly observed in patients with advanced cancer [28]. Although the precise pathogenesis of CC remains incompletely understood, the loss of muscle mass and function is considered the most critical clinical manifestation [29]. Despite extensive research, no effective clinical treatments for CC have been identified to date. Several potential therapeutic agents have been proposed, including growth hormone, insulin-like growth factors, and anti-myostatin agents [30,31]; however, current evidence suggests that these approaches do not offer sufficient efficacy in the treatment of CC [32].

The susceptibility to CC varies across different tumor types. CC is highly prevalent in patients with gastrointestinal and lung cancers, while it is less common in those with hematological malignancies or breast cancer [33]. In preclinical studies, various models of CC have been developed, including chemically induced models, tumor transplantation or orthotopic models, and genetically engineered mouse models [34,35]. In this study, we aimed to evaluate the therapeutic effect of phloretin on CC induced by different tumor types, using both C26 and LLC tumor-bearing mouse models. Our findings indicate that phloretin provides a protective effect against CC in both models. The rationale for utilizing these two distinct murine models was to validate the broad-spectrum therapeutic efficacy of phloretin across different genetic backgrounds and tumor-induced inflammatory profiles. While both are widely recognized as gold-standard models for CC, they exhibit marked discrepancies in their disease kinetics and systemic inflammation. The C26 model, established in BALB/c mice, is an acute and highly aggressive model primarily driven by massive systemic elevations of pro-inflammatory cytokines, particularly interleukin-6 (IL-6), which rapidly activates the STAT3 pathway in skeletal muscle [36]. In contrast, the LLC model in C57BL/6 mice typically presents a more protracted disease course. Its cachectic phenotype is associated with a different, relatively moderate systemic inflammatory profile compared to the acute IL-6 storm seen in C26 mice [37]. These distinct inflammatory milieus may also contribute to the observed variations in NF-κB signaling components across our models. Specifically, while phloretin consistently suppressed p-IκB, the response of p-p65 was more pronounced in the C26 model than in the LLC model. After confirming that total IκB and p65 levels were accurately normalized and remained stable, we attribute these subtle discrepancies to the highly dynamic and transient nature of NF-κB activation. IκB phosphorylation rapidly triggers its proteasomal degradation to permit p65 nuclear translocation; thus, our endpoint analysis might capture different kinetic snapshots of this continuous cycle depending on the specific inflammatory drive of each model. Although we did not directly quantify serum inflammatory cytokine levels in this study—which constitutes a limitation to be addressed in future investigations—our protein data demonstrate that phloretin effectively mitigated muscle atrophy in both the highly inflammatory C26 model and the more protracted LLC model. This underscores the robust potential of phloretin to counteract CC independently of the specific tumor cytokine milieu.

CC is a systemic metabolic syndrome driven by chronic inflammation. Tumors provoke host immune responses, triggering the release of inflammatory cytokines that subsequently lead to the loss of skeletal muscle mass and function [38]. It has been reported that in CC, cytokines such as interleukins and TNF-α [4], activate the STAT3 [39] and NF-κB pathways. Recently, a novel CC model based on IL-6 overexpression has been developed. This model recapitulates key clinical features of CC, including systemic inflammation, elevated plasma IL-6 concentrations, increased energy expenditure, and continuous loss of muscle and adipose tissue [40], underscoring the pivotal role of inflammatory factors and pathways. Our animal models consistently demonstrated activation of these pathways, and our results highlight that phloretin treatment effectively inhibited STAT3 activation, a key driver of muscle wasting.

The hallmarks of CC are skeletal muscle wasting and dysfunction. These two features are crucial for clinical research on CC [29]. In animal models, skeletal muscle characteristics are typically assessed by changes in muscle mass, myofiber cross-sectional area, and grip strength [41]. In the C26 model, phloretin significantly alleviated the pathological progression of CC, as evidenced by improvements in emaciation, fur condition, body weight, lean mass, along with a modest effect on grip strength. Furthermore, histopathological analysis revealed that phloretin attenuated adipose tissue depletion and skeletal muscle atrophy. Consistent with these findings, phloretin also exhibited protective effects in the LLC model, particularly in preserving muscle mass and fiber integrity, further supporting the efficacy of phloretin in mitigating tumor-induced muscle atrophy. Furthermore, phloretin treatment at 10 mg/kg/day did not adversely affect the body weight or behavior of healthy non-tumor-bearing mice, confirming the safety of this single-dose regimen for continuous administration.

Ubiquitination is a critical step in protein degradation [42]. The muscle-specific E3 ubiquitin ligases MuRF-1 (*Trim63*) and Atrogin-1 (*Fbxo32*) are key regulators of muscle atrophy associated with CC [43]. These genes regulate the degradation of myofibrillar proteins, leading to the loss of muscle mass and function [44]. Additionally, the activation of the ALS contributes to muscle atrophy [45]. While autophagy functions to maintain cellular homeostasis by clearing damaged organelles [46], excessive autophagy in CC contributes to skeletal muscle loss. In our study, both animal and in vitro models exhibited activation of the UPS and ALS. Phloretin treatment inhibited the activation of the UPS and ALS, preventing the reduction in MyHC. Our results align with previous studies suggesting that phloretin can inhibit specific pathways involved in skeletal muscle atrophy, including the STAT3 pathway, UPS, and ALS [6,14]. This suggests that flavonoids play a significant role in alleviating skeletal muscle atrophy induced by cancer cachexia.

Despite the promising findings, several limitations of the present study should be acknowledged. First, the C26 and LLC murine models utilized here represent acute and highly aggressive forms of cancer cachexia, which may not fully encapsulate the chronic and gradual disease progression observed in clinical scenarios. Second, our assessment of lean body mass relied on carcass weight—an oversimplified measure compared to gold-standard techniques such as DEXA or non-invasive body composition analysis. Third, the in vitro experiments employed a relatively high concentration of phloretin (100 μM). While this served as an effective mechanistic tool consistent with recent pharmacological studies evaluating STAT3 inhibition [47] to elicit robust molecular responses without cytotoxicity, it is unlikely to be fully achievable in vivo solely through standard dietary intake. Fourth, our evaluation of the autophagy–lysosome pathway primarily focused on steady-state protein levels of LC3B; thus, these results should be interpreted with caution as they may not fully reflect dynamic autophagic flux, which constitutes a limitation of the present study. Fifth, our in vivo experiments evaluated only a single dose of phloretin (10 mg/kg). While this established a clear proof-of-concept, future studies incorporating a comprehensive dose–response gradient are required to identify the optimal therapeutic window. Finally, although phloretin showed a protective trend, its effect on functional recovery, such as grip strength, did not reach statistical significance in the LLC model and was only modestly significant in the C26 model. Furthermore, we did not systematically quantify the daily food intake of the mice due to group-housing conditions. Consequently, we cannot fully exclude the potential influence of altered food consumption on the overall body weight changes, which remains a factor to be investigated in future individually housed studies.

In summary, our study demonstrates that phloretin treatment alleviates the loss of skeletal muscle mass and function, as well as the depletion of adipose tissue, in the context of cancer cachexia. The underlying mechanism primarily involves the inhibition of STAT3 pathway activation, which subsequently suppresses UPS and ALS activation, downregulates the expression of MuRF-1 and Atrogin-1, and thereby protects against skeletal muscle loss while restoring muscle mass and function. Therefore, phloretin represents a promising candidate for the treatment of cancer cachexia.

## 5. Conclusions

**Figure 6:**
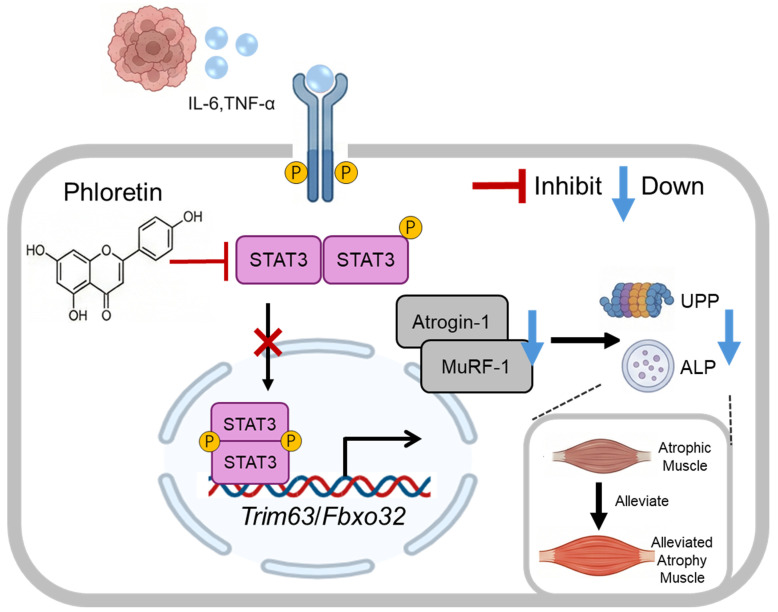
Schematic representation of the molecular mechanisms by which phloretin mitigates cancer cachexia-induced skeletal muscle wasting. Tumor-derived inflammatory cytokines (e.g., IL-6, TNF-α) trigger the phosphorylation and activation of STAT3 in skeletal muscle cells. Activated STAT3 upregulates the transcription of muscle-specific E3 ubiquitin ligases, *Trim63* (MuRF-1) and *Fbxo32* (Atrogin-1). This subsequently hyperactivates both the ubiquitin–proteasome pathway (UPP) and the autophagy–lysosomal pathway (ALP), leading to massive protein degradation and muscle atrophy. Phloretin treatment effectively inhibits the phosphorylation of STAT3, suppressing the downstream expression of MuRF-1 and Atrogin-1. Consequently, the aberrant activation of UPP and ALP is downregulated, ultimately alleviating cancer cachexia-induced skeletal muscle wasting.

In conclusion, this study demonstrates that phloretin effectively ameliorates cancer cachexia-induced muscle wasting in both in vivo and in vitro models, although its efficacy in restoring muscle function requires further investigation. Mechanistically, phloretin exerts its protective effects by targeting STAT3 signaling, thereby suppressing the activation of the UPS and ALS (illustrated in Figure 6). These findings highlight the potential of phloretin as a promising therapeutic agent for the clinical management of cancer-associated cachexia.

## Data Availability

The original contributions presented in this study are included in the article. Further inquiries can be directed to the corresponding authors.

## Supplementary Materials

The following supporting information can be downloaded at: https://www.mdpi.com/article/10.3390/biomedicines14051004/s1. Figure S1. Effects of phloretin on inguinal white adipose tissue (iWAT) mass, heart mass, tumor mass, mean food intake, and the ratio of lean mass to initial weight in cachectic models. (A)The iWAT mass in each group of C26 cancer cachectic mice (BALB/c). (B) Heart mass in each group of C26 cancer cachectic mice (BALB/c). (C) Tumor mass in each group of C26 cancer cachectic mice (BALB/c). (D) Mean food intake in each group of C26 cancer cachectic mice (BALB/c). (E) The ratio of lean mass to initial weight in each group of C26 cancer cachectic mice (BALB/c) (*n* = 6 per group). (F) iWAT mass in each group of LLC cancer cachectic mice (C57BL/6). (G) Heart mass in each group of LLC cancer cachectic mice (C57BL/6). (H) Tumor mass in each group of LLC cancer cachectic mice (C57BL/6). (I) Mean food intake in each group of LLC cancer cachectic mice (C57BL/6). (J) The ratio of lean mass to initial weight in each group of LLC cancer cachectic mice (C57BL/6). *n* = 6 per group. The data are presented as the mean ± SEM. One-way ANOVA followed by Tukey’s post hoc test was used. Statistical significance: ns means no significance; * *p* < 0.05; ** *p* < 0.01; *** *p* < 0.001. Figure S2. Quantitative analysis of the relative expression levels of total STAT3, IκB, and P65 proteins. The protein expression levels of total STAT3, IκB, and P65 were quantified and normalized to GAPDH. Data are shown for the skeletal muscle of C26-induced cachectic BALB/c mice (left panel), LLC-induced cachectic C57BL/6 mice (middle panel), and C26 conditioned medium (CM)-induced C2C12 myotubes (right panel) following phloretin treatment. The data are presented as the mean ± SEM. One-way ANOVA followed by Tukey’s post hoc test was used. Statistical significance: ns means no significance; * *p* < 0.05. Table S1. Detailed information of the primary antibodies. Table S2. Detailed information of the secondary antibodies.

## Abbreviations

The following abbreviations are used in this manuscript:

C26	Colon26
LLC	Lewis lung carcinoma
CC	Cancer cachexia
NF-κB	nuclear factor-kappa B
ALS	Autophagy-Lysosomal System
Atrogin-1	Muscle Atrophy F-box
CSA	Cross-Sectional Area
GAS	Gastrocnemius Muscle
iWAT	Inguinal White Adipose Tissue
MyHC	Myosin Heavy Chain
UPS	Ubiquitin–Proteasome System
P62	Sequestosome 1
GDF-15	Growth Differentiation Factor 15
TNF-α	Tumor Necrosis Factor-alpha
STAT3	Signal Transducer and Activator of Transcription 3
MuRF-1	Muscle RING-Finger Protein-1
