# Supplementary material for: Phloretin Attenuates Cancer Cachexia-Induced Skeletal Muscle Wasting Associated with the Modulation of STAT3 Signaling
Source: Biomedicines. 2026 Apr 28;14(5):1004. doi: 10.3390/biomedicines14051004 (PMC13204840; doi:10.3390/biomedicines14051004)
Supplement: Supplementary file 1 [file biomedicines-14-01004-s001.zip › biomedicines-4244723-supplementary.pdf]

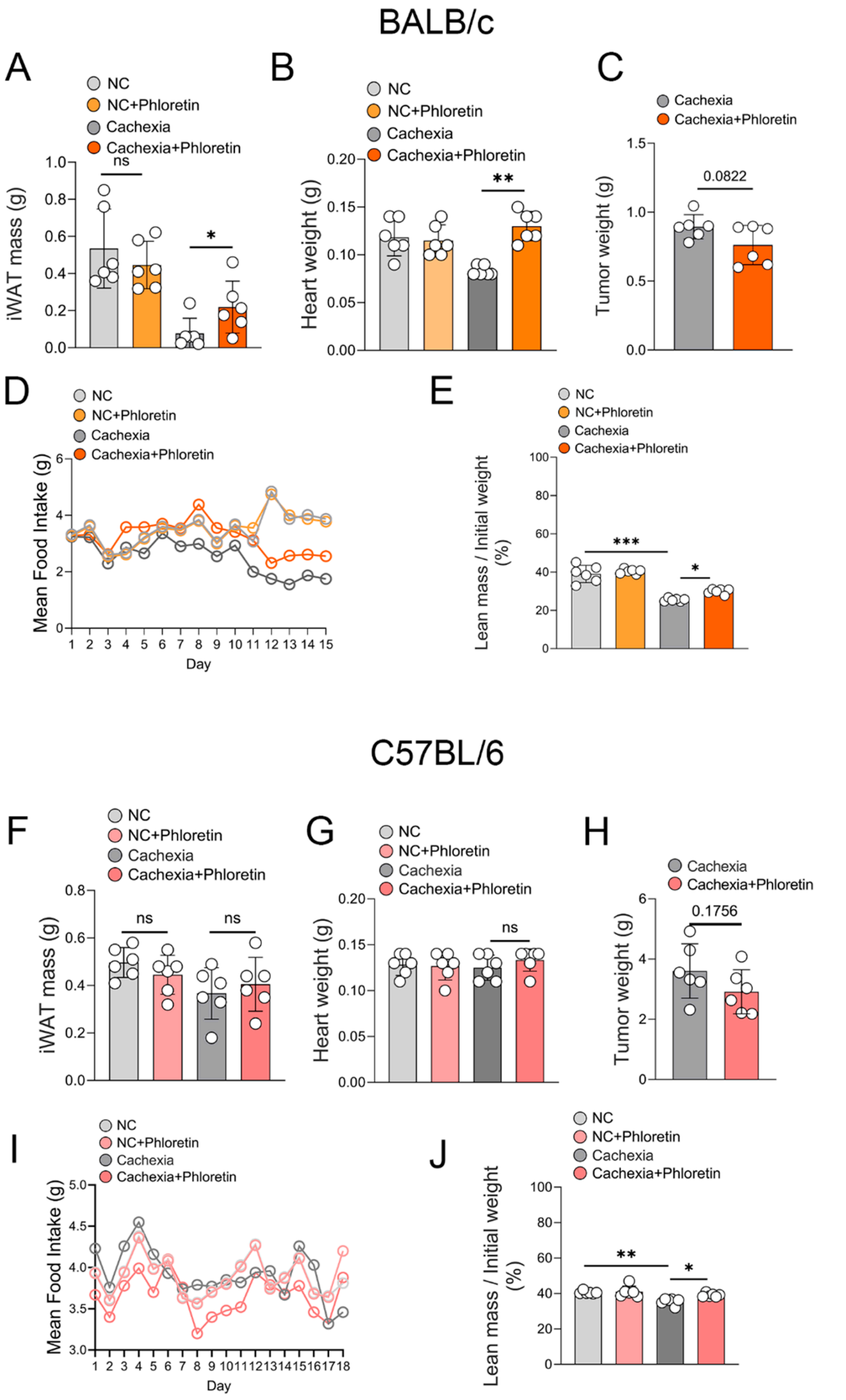

Figure S1. Effects of phloretin on inguinal white adipose tissue (iWAT) mass, heart mass, tumor mass, mean food intake, and the ratio of lean mass to initial weight in cachectic models. (A)The iWAT mass in each group of C26 cancer cachectic mice (BALB/c). (B) Heart mass in each group of C26 cancer cachectic mice (BALB/c). (C) Tumor mass in each group of C26 cancer cachectic mice (BALB/c). (D) Mean food intake in each group of C26 cancer cachectic mice (BALB/c). (E) The ratio of lean mass to initial weight in each group of C26 cancer cachectic mice (BALB/c) (n=6 per group). (F) iWAT mass in each group of LLC cancer cachectic mice (C57BL/6). (G) Heart mass in each group of LLC cancer cachectic mice (C57BL/6). (H) Tumor mass in each group of LLC cancer cachectic mice (C57BL/6). (I) Mean food intake in each group of LLC cancer cachectic mice (C57BL/6). (J) The ratio of lean mass to initial weight in each group of LLC cancer cachectic mice (C57BL/6). n=6 per group. The data are presented as the mean ± SEM. One-way ANOVA followed by Tukey's post hoc test was used. Statistical significance: ns means no significance; *$p < 0.05$; **$p < 0.01$; ***$p < 0.001$.

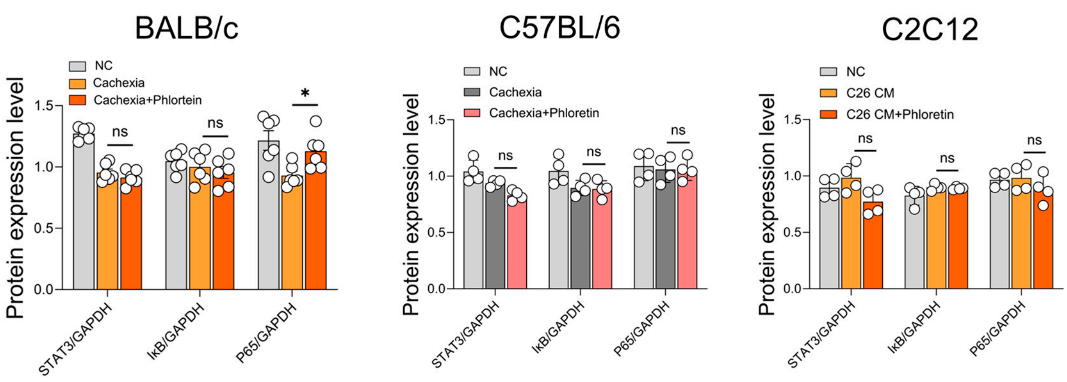

Figure S2. Quantitative analysis of the relative expression levels of total STAT3, IκB, and P65 proteins. The protein expression levels of total STAT3, IκB, and P65 were quantified and normalized to GAPDH. Data are shown for the skeletal muscle of C26-induced cachectic BALB/c mice (left panel), LLC-induced cachectic C57BL/6 mice (middle panel), and C26 conditioned medium (CM)-induced C2C12 myotubes (right panel) following phloretin treatment. The data are presented as the mean ± SEM. One-way ANOVA followed by Tukey's post hoc test was used. Statistical significance: ns means no significance; * $p < 0.05$.

Table S1. Detailed information of the primary antibodies.

Primary Antibodies	Catalog Number	Manufacturer	Application	Dilution Factor
p-STAT3	#9145	Cell Signaling Technology	WB	1:1000
STAT3	#12640	Cell Signaling Technology	WB	1:1000
GAPDH	AF0006	Beyotime Biotechnology	WB	1:5000
p-IκBα	AF1870	Beyotime Biotechnology	WB	1:1000
p-P65	AB3013	Beyotime Biotechnology	WB	1:1000
P65	AF1234	Beyotime Biotechnology	WB	1:1000
IκBα	10268-1-AP	Proteintech	WB	1:1000
LC3	14600-1-AP	Proteintech	WB	1:1000
P62	18420-1-AP	Proteintech	WB	1:1000
MuRF-1	sc-32920	Santa Cruz Biotechnology	WB	1:500
Atrogin-1	sc-166806	Santa Cruz Biotechnology	WB	1:500
MyHC	MAB4470	R&D Systems	WB	1:1000

Table S2. Detailed information of the secondary antibodies.

Secondary Antibodies	Catalog Number	Manufacturer	Application	Dilution Factor
Peroxidase-conjugated anti-mouse	FDM007	Fudebio-tech	WB	1:50000
Peroxidase-conjugated anti-rabbit	FDR007	Fudebio-tech	WB	1:50000
Fluorescence-labeled anti-mouse	A0521	Beyotime	IF	1:1000
